# Mr. Pring on the General Indications Which Relate to the Laws of Organic Life

**Published:** 1820-12-01

**Authors:** 


*General Indications which relate to the Laws of the Organic Life.* By Daniel Pring, Member of the Royal College of Surgeons, London. One Vol. Octavo, pp. 352, London, 1819.

"———— presume not God to scan."—*Essay on Man.*

One prominent and leading principle of the work before us is to demonstrate the non-existence of a "Great first Cause" —of a God that created us—of a Providence that rules over us.

"Hence it follows that the principle of intelligence, this same designing principle, must be made by causes, or *is their effect;* and as the causes individually must be different from the effect, so there were agents which preceded design, and without the guidance of design formed the designing principle itself: and yet it is said that nothing good, excellent, or regular, can be produced without design; while it must be admitted, agreeably with natural evidence, that design itself must have been produced without it.

"According then to the preceding principles, which are merely exhibited as a sketch of the indications of nature, the following is the state of the question with respect to the influence of a pervading *intellect,* or universal mind:

1. "This visible world cannot have been produced *de novo,* not having existed in any form before, by mere intellectual influence; since no cause can supply that which it does not possess, or be either more or less than itself, or contribute any other influence than that which is comprised in its own existence.

2. "Such universal mind itself must have been produced by its causes; and these latter, determining all effects, determining all operations (since nothing can take place but by them), must govern such universal mind, making it that *which it is*, and making it in its turn concur in the general scheme of causation." 43.

Thus what we, poor ignorant mortals, have hitherto considered as an Omnipotent, Omniscient, and Omnipresent Deity, who constructed the universe—and still preserves it,

"Principium, Rector, Dux, Semita, Terminus idem,
"Qui regit immensam justo moderamine molem,"

turns out to be only a blind sub-agent, formed himself by anterior, and consequently more powerful causes, which govern and determine all his operations—making him "in his turn concur in the general scheme of causation!"

We never had a doubt that atheism would be the end of materialism. When men presume to determine on what are, or what are not, *final causes*, (a subject completely beyond the reach of human comprehension) the natural consequence is, what the foregoing extract has unequivocally pourtrayed. Now, as we consider that the great laws and ties, by which society are held together in peace and comparative happiness, depend mainly for support, on a belief, not only in the existence of a Deity, but in his superintending providence over the universe,

"Qui pelagus fluitare jubet, consistere montes,
"Qui corpus mentemque dedit ——————"

so we think that the man who endeavours to destroy this belief, commits a greater crime against the public good, however innocent his intentions, than he who goes about to sap the foundation of human laws and political institutions.

If it be said—and it is here said—that these philosophical doctrines are only designed for philosophers, and will not be read, or if read that they will not be understood, by the vulgar; we reply that, in the present state of knowledge and its facility of diffusion through all classes of society, the tenet of the philosopher, when it suits the purpose, is quickly converted, by the licentious, the seditious, and the unprincipled leveller, into a powerful engine of corruption for the discontented and ignorant orders of the community. When therefore the surgeon, with his scalpel in hand, boldly proclaims the non-existence of a soul, and the metaphysical physician, by an elaborate process of reasoning, demonstrates the non-existence of a Deity, can we wonder that the active radical philosophers of the day should avail themselves of these advantages, by familiarizing the abovementioned doctrines for popular comprehension, and disseminating the most fatal poison through the veins of the body politic? That this is positively the case, every man of common observation may be convinced by the slightest glance at the state of things around him. And granting, for a moment, that this were not the case, to the extent described, are we authorized to lay before the junior branches of medical society a system of atheism and fatalism, which unequivocally annihilates every restraint upon the passions and conduct of man? If the philosopher's deity himself be but a blind agent in the "general scheme of causation," obeying implicitly and unconsciously the operation of causes "which make him what he is," can MAN be then a moral or responsible agent, more independent than his God? No! the actions of his life are the necessary *effects* of causes over which he has no control. To take a neighbour's life, or seduce a neighbour's daughter, therefore, can no more be a crime, than to feed the hungry or clothe the naked can be a virtue! Let fathers, husbands, and brothers, peruse with attention the following extract.

"It has been asked, first, if there were no such universal *mind*, how came we to have the notion of such a thing? It has been further inquired, secondly, how the notion of an intellectual and moral presence *came to be so prevailing*, that there is perhaps no people who have not the idea of some powerful and intelligent being who governs the world? *For the first question, it is sufficient to remark, that there are such things as fictions*, and in the same manner as they arise might originate any notion, and consequently the one we are considering; the notion is formed by the combination of ideas from sensible impressions, and by the assumption of analogies. Thus much is sufficient to shew that the *origin* of a notion is no proof of its truth, for by the same processes we are originating notions, some false and some true, every day. And for the second question, it is only to be observed, that if a fiction is one to which human nature, from similarity of constitution, is prone, *it will be very likely to be an universal one, without being the less a fiction.* Thus in the more ignorant times, and now among ignorant nations, spiritual agencies were perpetually occurring in nature, and affecting the concerns of men: the influence of the planets over certain affairs has likewise been a prevailing fiction, which however is discredited by men of sense and reflection, because they find no evidence for the opinion; and without this support it is not consistent with their character to fill their understandings with bad conceits, when the value they set upon good ones is shewn by the pains they take to find them. Thus much for the questions: I proceed to shew that the belief of some universal governor is one which men must be prone to slide into by an easy gradation from the common observance of causes.

"The notion of such an existence might arise out of the almost intuitive assent to a principle which has hitherto appeared to be the basis of a different argument: the principle is this, that nothing can exist without a cause; and now for the application. It is observed, that things do not make themselves: this observation disposes us to look for their causes. As one instance, we will take a human bone (or any other bone,) but, for the sake of precision, a human tibia: what produces it, what constitutes it? It will be replied, a bone is made by the union of phosphate of lime, phosphate of magnesia, carbonate of lime, sulphate of lime, gelatine, fat, and cartilage; blood-vessels, &c. exist in bone, and our tibia has a certain arrangement. Now urge the question further, which is very natural, and ask what determines this arrangement? We are not acquainted with the agents, and therefore supposing the necessity of a cause, as observed above, we say, God. Again the phosphate of lime, if first detected in bones, would be considered a simple substance: if it should, during this opinion, be asked how this phosphate of lime came to be? still retaining the necessity of some cause, it would be replied, it was created by God. But another step of analysis would give rise to a different reply; for when the two materials of which this substance consisted were known, the question, how it came to be, would be answered, "by the union of lime and phosphoric acid." Every example tends to shew that a Divine agency is assigned to begin where analysis, or the knowledge of causes, ends. Thus it happens that the assigned extent of the influence of the Deity is absolutely abridged as science advances; for as known causes are developed, the unknown cease to be supposed. In this way then the idea of some general antecedant cause comes to be obtained, and it is founded upon the acknowledged necessity of such a cause, *which necessity must obtain equally* (or the necessity is limited without reason, and in a case in which the universal analogy holds good) *with respect equally to the existence of the Deity as of other things;* this is a point which has been discussed.

"The notion of a first cause being in this way acquired, men very soon and very naturally extend their imaginations, and they next conclude him to be a *moral agent.* This conception is as easy as the other, and equally fitted to become prevalent, without natural evidence of the stricter sort. Thus, all those things by which we are liable to be affected, are related with us in such a way as to produce either agreeable or disagreeable sensations: the causes of the former we call good, those of the latter, evil. Now who dispenses these? Why, no other than the first cause which made them to exist. Then we come to invoke this cause to bestow upon us what is good, and remove that which is evil, *and it is very seldom that we are gratified,* except as an ardent desire for the possession of an object induces us to make strenuous efforts to obtain it. *Deities are in this way formed by the personification of causes,* and, as in the Mythology, a particular God may be assigned to each department of causes." 45.

Before making any remarks on the blasphemous impiety of the foregoing conclusions, we shall set before our readers a specimen of the reasoning whence they are drawn.

"What is a *cause?* The term implies a relation: it is that which is capable of producing something different from itself, which something is called an *effect.* This also is a relative term, it implies that which results from the operation of a cause.

"What virtue is there in a cause which enables it to produce something different from itself? a question well urged: why truly, none. If a cause could produce an effect which is different from itself, that in which the difference consists, if it be superadded to the cause, must originate from non-entity: which is contrary to our established principle. If it be only a part of a cause, some properties having been abstracted, why then there is no act of production; for that only remains, and is the effect, which was before produced. A single cause is no agent, it is an identity, but capable of no transaction: for a thing cannot supply or confer what it does not possess; all it can supply is itself, or its own identity. How then do effects comprise the cause, and still be something different from the cause?

"As a single cause can produce nothing different from itself, and as the effect, according to the relative signification of the word, is more than the cause; and as this difference cannot originate out of non-entity; so the difference must on these accounts be supplied by something else. The effect then depends, not upon one, but upon more than one cause; and as all things are effects, there can be nothing simple and elementary, but all things must be produced by *causes.*

"The causes which make an effect can supply nothing but themselves, nothing but that which pre-existed; the effect therefore is no new existence, but it is a new form; called *new*, from its having taken place at some known period. Surely, it will be said, an effect appears to be very different from its causes? It must be different from its individual causes, but *is* that which it is made by them all; *the causes must be different individually from their effect, or the whole;* and this, in some respects, is a gigantic principle.

"The mode by which a cause acts has nothing mystical in it, *it is itself, and no more than itself, and it can do no more than exist.* But it may exist separately, that is, as an effect dependent only upon its own causes. When it performs that which characterizes a cause, viz. when it produces an effect, it is by combining with something else.

"In this combination there is no new production; both causes (or if they were a thousand it would be the same thing) are changed, so as to exist, still preserving every property which belongs to them, in another form. This other form is the effect which is thus conjointly produced.

"Causes do not lose their existence in changing their form: although in the effect the causes separately may not be recognized; they cannot lose their existence; the *whole* is a different combination, and of course possesses a double set of properties, which will individually have some share in determining its character. Every thing is liable to be considered as an effect: and all those things are liable to be considered as causes, which, from a relation between themselves and others, might change their form and produce effects." 21.

Such is the ratiocination of a medical philosopher, whose Pythian ravings have been represented by some of our cotemporaries, as the sublimest truths that ever issued from the human mind—the emanations of a genius incomparably superior to that of a Homer, a Milton, a Locke, or a Shakespeare! Now to our humble comprehension these sublime effusions appear to be the dullest mass of unintelligible jargon —the wildest chaos of addle-headed lucubrations ever engendered in the brain of man or woman, excepting on the tripod at Delphi, or in the cells of a mad-house.

We have lately heard an intelligent physician remark that the obscurity or rather unintelligibility of the reasonings would effectually prevent the work from being read, and consequently obviate its dangerous tendency. We cannot altogether agree in this remark. Many who cannot understand the *arguments*, may be staggered by the boldness of the *conclusions;* and we all know that obscurity itself too often passes in this world for depth of understanding and thought. On this account we deem it our duty to point out the absurdity of the reasonings and the danger of the deductions; although we are well aware that the Atheistic Phalanx will shower on us the titles of hypocrites, fanatics, enthusiasts, &c. for these liberal philosophers will not allow that a particle of honesty, candour, or independence of sentiment, can possibly exist beyond the limits of their own sect! Let us, however, examine how far *they* observe the strict line of candid and ingenuous conduct. The long extract which we laid before our readers a few pages back has given them some notion of what Mr. Pring means by the term Deity—namely, an item "in the general scheme of causation," "produced by its *causes*, and *these latter* determining all effects, and *governing such universal mind*, making it what it is." Now we need not tell our readers that we look upon the Deity as a very different being—namely, as an omnipotent, wise, and designing Providence that made the universe and still governs it. What will be thought of the consistency and candour then of a man, who, after ranking the Almighty with a Chinese joss, a mythological fiction, a blind sub-agent, or link-god, in the general chain of causation, dexterously shields himself, at the conclusion, from the charge of Atheism by the following declaration of his creed.

"I profess myself (as the nature of my profession may not by some be understood) with *all sincerity* to be a firm believer in the existence of *a* Deity; and I ascribe to this Deity, even on physical testimony, *perhaps* all that revelation strictly requires."* 58.

Thus Mr. Pring either disbelieves all that he had formerly laboured to establish, or in the words "*a* Deity," and "*perhaps*" he has left himself a mental reservation by which he hopes to seem one thing and be another. That we have neither misunderstood nor misrepresented him we can prove by an appeal to the testimony of his professed admirer Dr. Hutchinson, who, in his last proemium, distinctly states that Mr. Pring's system is "*unfavourable to theology.*" How Mr. Pring and his advocates will explain this contradiction or equivoque we know not; and how often they may chuse to thunder forth their epithets of hypocrisy and dissimulation against us, we care not.

But we have yet another charge to prefer against Mr. Pring, in which he first outrages our feelings, and then insults our understanding. After his laborious attempt to establish Atheism, and consequently subvert Christianity, he coolly tells us that all these PHYSICAL proofs of Atheism have nothing to do with religion, since, "*Christianity sets Nature aside, and entirely out of the question:* it is proposed by its author that it should be accepted in the way of *confidence*, or trust upon his authority; and whether *natural testimony concur with or oppose the system*, is a point which does not at all affect the grounds or terms upon which it is *recommended.*" 57. Thus our author contemptuously leaves it to credulous Christians to believe—"*quia* impossibile*!*" Mr. Pring knows less of the material world in which he resides, than of the metaphysical world into which his imagination has wandered, if he thinks such insults to common sense, as the above passages convey, can raise him in the estimation of any man whose opinion is worth consulting.

And here we beg to call the attention of our brethren to the now prevalent system of amalgamating metaphysical and atheistical doctrines with medical and surgical subjects. The faculty has already suffered, in some degree, by the indiscretion of a few of its members; and we conceive that if we value our own respectability, or the esteem of society at large, we should check and discountenance these licentious aber-

---

* In the very page before this he gives a flat contradiction to this "physical testimony;" for he says:—"The true and *only* source of proof, by which the *existence and moral government of the Deity* can be consistently established, is revelation." 57.

rations of the prurient imagination, ere it be too late. We live in a period when the harsher features of the human mind are rapidly developing themselves—when the moral restraints over the passions are bursting asunder, one after another—when divine laws are contemned, and human laws resisted—when physical force is openly opposing itself to moral feeling—and when nothing but the fear of punishment can restrain the commission of crime. At such a period, shall those, whose duty and employment it is to alleviate the sufferings of humanity, be found aiding in the work of demoralization, by organizing fundamental principles for radical systems of revolution and ruin! We have selected, as our motto for this article, the memorable words of the poet—"presume not God to scan!" It is not beyond the range of possibility, nay, of probability, that a licentious indulgence in the fruit of that tree of knowledge, which once before—

"Brought death into the world, and all our woe,"

may yet again precipitate man from his Eden of civilization and science into the gulph of barbarism and ignorance! All extremes, like great and heavy bodies, are endued with potent principles of approximation. Tyranny, under our own eyes, has risen, Phœnix like, and clapt its gloomy wings over the ashes of Liberty consumed by her own *excessive* fires. Where is the bright and too often impious intellect of Greece? A crescent only of her former orb of light remains; the badge of her degradation and emblem of her mental night! Where is the thunder of the Roman senate? Changed to a mummery over beads and crosses!—Even in this country, the Press, that pride of art, that palladium of freedom, that universal organ of thought, whose voice is heard wherever earth extends, may yet prove the engine of its own destruction! Good and evil are its products; and should the latter come to predominate, the event may be readily imagined.

In the mean time, we conceive it to be the duty of the medical profession, and particularly of medical writers, not only to stand aloof from the ranks of impiety, but to raise their voice in support of religion and virtue.

And here we cannot but lament that such a man as Mr. Pring, whose talent is great, though wrongly directed, because employed on subjects beyond the reach of human intellect, should waste his mental energies on dangerous, or, at the best, *useless* metaphysics, when the practical part of his profession offers such a wide field for cultivation. If the multitude of *physical* subjects yet unexplored, can afford him no hope for the successful exercise of his talents, what prospect can he have in fields of airy speculation?

"Missis igitur hujusmodi commentis de rebus quas Natura forsitan visibus humanis negaverit, tanquam ad *inutilia et incomprehensibilia, vel absurda fortasse*, ducentibus, magis e re sua erit, si medici ad singulas res factas et veras de hoc argumento investigandas semet graviter accinxerint." *Gregory.*

Even Sydenham, than whom no man was ever fonder of a bit of abstruse theory, acknowledges the absurdity of prying into *first causes*. "Nimirum certissimis ubique legibus, ac artificio sibi soli intellecto, rerum omnium generationes natura parens exsequitur; quoque uspiam e causarum gremio in actum, ac quasi in lucem, educit, eorum essentias, quidditates, ac differentias constitutivas, altissimis tenebris obvelat." P. 110.

Mr. Pring asserts that the Deity himself can make nothing where nothing previously existed. Now according to this very rule our author's labours must be abortive; for as his, or any other human being's, knowledge of the nature of God amounts to NOTHING, so *nothing* can result from this investigation—save only a book.

We are not, at the same time, blinded by prejudice; for we can plainly perceive, and we freely acknowledge, that the work before us, even in its metaphysical portions, contains unequivocal indications of a powerful mind. There are some passages too, exceedingly eloquent, and one of the most beautiful we shall here insert. It is put into the mouth of an *enthusiast*:—

"Great Nature, by whatever name expressed, it is to thee I address myself! thee I contemplate! thou art my theme: but where begin to think, where begin to speak of thee! I view, at night, a large expanse of hill and dale, shaded with trees, clad in luxuriant verdure; or naked, sighing at the rude attacks of wintry blasts. Imagination paints the extent beyond, where earth is mottled by other shapes and clothing; with other animals to enjoy her fruits. From this terrestrial scene, the view ascends to those revolving orbs, this lofty dome, adorned with stars and planets. These things I contemplate, and wonder, Nature, at the vastness of thy space and works; thy silence breathes into my soul; all is immensity, engendering wonder. Yet this first impression once abated, a speck of thy production, with faculties, the offspring of thy bounty, presumes to scan thy methods, and pry impertinently into ways which thou hast studied to conceal. But forgive the trespass, it is love of thee that prompts this curious zeal, and guides my thoughts astray; it is thy work, that they should adore thee; take it, therefore, not amiss, that falling from the amazement which is first inspired by thee, I seek to know at least thy scheme, though ignorant of thy means, thy instruments, and subtler agencies.

"Thy movements give birth to time, yet thy existence acknowledges no period; thou hast made time, and wilt not be obedient to thy creature: we boast some records of thy existence, and presume to fix a date to thy beginning; but if *then* thou didst *commence*, from whence derived? or how start forth from nothing? Thy own nature, thy inherent and proper forces, had no share in thy origination, for that would be to date thy actions previous to thy birth. How then didst thou begin? Methink, the spirit of the hills, at the question, shakes from him his beloved repose; himself a part, speaks with a commissioned voice the language of the whole; yet it is a voice sweet and soft, it floats like a zephyr, and is heard only in the stillness of the world; it is a whisper to the soul, which swells when it comprehends the great idea, and echoes thus the truth, in accents of its own: "Search not when that began which always has been: ages and ages have revolved, myriads of changes have been wrought, forms have been made, endured, and vanished; destruction has succeeded quickly to creation; yet Nature was, before all this; her processes were repeated in periods infinite, which thou, with a capacity for finite purposes, understandest not, but must still think true."

"Is then great Nature indebted to no other power but her own? Say what this other power is, and try if here our thoughts of *infinite duration* succeed more happily; *something* had no beginning then; the voice is surely no chimera: hark! it speaks again.

"What in this world, which so excites thy admiration, canst thou perceive but an assemblage of forms? Thou wouldst know when and how they came to be. Oh! dull perceiver! little dost thou deserve to rise to universal truths, if thou so readily canst overlook what in thine own experience is without exception. Observe of things which *are*, but *were not:* thyself observe. The sun has not yet thirty annual courses run, since the creatures which are like thee knew thee not; ask how thou camest to be, what has produced the thing thou art? Thy history is clear: thy formation has, throughout, been passive; that which thou hast, by which thou dost exist, is given thee; nought hast thou but what is conferred; conferred by whom, or what? A form thou hadst prepared for change, from others like thyself derived, but most imperfect. And what made this? thy curiosity would ask. It is plain, an assemblage of existences, of occult forms or properties, whose being is inferred, because *existence* is their effect, but which to develop will yet for centuries to come make full employment for the restless spirits of thy kind. But this imperfect form derived, the earth feeds with constituents, animals, and plants: these transfused supply thy growth with its materials, and thy accessions are as they are furnished."

"Yes, this is the manner of it: existences all related; and their relations fixed, not by themselves, but by the force of the existences which are included within themselves; *existence still maintains existence, and nought begins where no existence is.*

"What sum of admiration is sufficient for this grand world, enclosing in itself an endless series of forms and combinations! Existence still springing from itself, and by itself perpetuated; whose beginning no time has witnessed, whose end no period will define; existing without our knowledge how; describing various shapes, pursuing various changes, none occurring but existence still compels; all enduring in their present, or in other forms, *because existence has no power to be nothing.*

"The stars are yet upheld: great bulks we must acknowledge them, apparently above us: and they fall not though propped only by light etherial columns. Who shall say why they keep their spheres? who shall say what they are; whether constant, or at periods produced by agents which we do not know, in worlds teeming with things and processes of which we here find no examples? They govern not themselves, but are obedient to their own constituents; there, too, existences are causes, and all we contemplate in them is yet compelled, *effected* by existence.

"The sun is present, and imparts to us both light and heat; it is formed by its own causes: these, or its grosser forms, by others, an endless chain. In turn it sends to us some causes, which it well can spare: to us it sheds existence, which mingles with our substances and creates new forms.

"The sea possesses by a natural right, the deep dominions over which it rolls. This vast property it claims by force of causes which with it abide; it seeks the lowest parts, and terrifies its confines all around by bold incursions on the soil which man calls his; it foams and dashes against great rocks, a bulwark formed to check its aggrandizing spirit, and make its waters still recoil upon itself. Fruitless ambition! thy powers have but their scope; and further, earth is too mighty for thee, as it, in thy dominions, and all its fine productions, are but a weakness, serving for thy pastime.

"Myriads of waves roar and froth, or, gliding smoothly, glitter in the sun upon thy melting bosom. Not one of these that moves, but moves as 'tis impelled; it, passive, an effect; in turn impelling, then a cause; all more minutely propertied; each particle which we suppose, but cannot see, of the same quality with the whole: fluid and salt; one while upbearing, then yielding; at one time pleasant and salutary for those of a different element, at another, threatening, overwhelming, and destructive; now, transporting rich freights in safety to the shores, dispensing wealth and luxury, then swallowing without remorse, this merchandize (the sovereign curse of nations), and bringing ruin, as indeed is just, on those who rest their hopes and fortunes on such trash! Thou, too, great sea! endless in thy relations.

"Thy movements observe a method even in their roughness: one while thy waves overstep their present limits, the pebbles on the strand are seen no more, thy presence hides them, and they chafe and fret, obscurely warring with each other, where no witness is to tell the fate and history which must belong to each. They, by their causes, are, where we observe them, still passive; they are removed, or broken, or rest, or remain a whole; or are collected, some fused, some ground on roads and then manure the fields; or else are washed along, now backwards, then onwards again, traversing the uneven bottom of the deep from shore to shore. Again the waves retire, the sand is wet, the sun compels from it a contribution, and it is dry; these changes regular, flux and reflux, marking periods, and obedient to some other power. What other power? The moon, they say; but this is perhaps a fable, coincidence is not causation; still by *some* power governed in this regular work; again connecting the great sea with unknown things, an endless series of relations." 51-2-3-4.

Some apology may be necessary for the length of this and some other extracts—indeed for the introduction of a metaphysical article at all, into a journal devoted to practical medicine. It is very seldom that we shall be guilty of such trespasses on our general plan; but the nature of the subject may plead our excuse on this occasion. In our next number, we shall, probably, take up the physiological part of the volume, which may be more to the purpose. In the mean time, we entreat Mr. Pring to cooly reflect on what he has done, and what we have said. The events of the next twenty years may furnish, perchance, a memorable commentary on both.

